# Involvement of Acetylcholine Receptors in Cholinergic Pathway-Mediated Protection Against Autoimmune Diabetes

**DOI:** 10.3389/fimmu.2019.01038

**Published:** 2019-05-15

**Authors:** Maria J. Fernández-Cabezudo, Junu A. George, Ghada Bashir, Yassir A. Mohamed, Alreem Al-Mansori, Mohammed M. Qureshi, Dietrich E. Lorke, Georg Petroianu, Basel K. al-Ramadi

**Affiliations:** ^1^Department of Biochemistry, College of Medicine and Health Sciences, United Arab Emirates University, Al-Ain, United Arab Emirates; ^2^Department of Medical Microbiology and Immunology, College of Medicine and Health Sciences, United Arab Emirates University, Al-Ain, United Arab Emirates; ^3^Department of Cellular Biology and Pharmacology, Herbert Wertheim College of Medicine, Florida International University, Miami, FL, United States

**Keywords:** cholinergic stimulation, acetylcholine, muscarinic AChR, neuroimmunology, type I diabetes

## Abstract

Type I diabetes (T1D) is a T cell-driven autoimmune disease that results in the killing of pancreatic β-cells and, consequently, loss of insulin production. Using the multiple low-dose streptozotocin (MLD-STZ) model of experimental autoimmune diabetes, we previously reported that pretreatment with a specific acetylcholinesterase inhibitor (AChEI), paraoxon, prevented the development of hyperglycemia in C57BL/6 mice. This correlated with an inhibition of T cell infiltration into the pancreatic islets and a reduction in pro-inflammatory cytokines. The cholinergic anti-inflammatory pathway utilizes nicotinic and muscarinic acetylcholine receptors (nAChRs and mAChRs, respectively) expressed on a variety of cell types. In this study, we carried out a comparative analysis of the effect of specific antagonists of nAChRs or mAChRs on the development of autoimmune diabetes. Co-administration of mecamylamine, a non-selective antagonist of nAChRs maintained the protective effect of AChEI on the development of hyperglycemia. In contrast, co-administration of atropine, a non-selective antagonist of mAChRs, mitigated AChEI-mediated protection. Mice pretreated with mecamylamine had an improved response in glucose tolerance test (GTT) than mice pretreated with atropine. These differential effects of nAChR and mAChR antagonists correlated with the extent of islet cell infiltration and with the structure and functionality of the β-cells. Taken together, our data suggest that mAChRs are essential for the protective effect of cholinergic stimulation in autoimmune diabetes.

## Introduction

Type 1 diabetes (T1D) is an autoimmune disease characterized by the progressive destruction of the insulin-producing β-cells in the pancreatic islets of Langerhans by autoreactive T cells. The development of the disease starts with the β-cell destruction in individuals with genetic pre-disposition and under specific environmental factors, followed by recruitment and activation of inflammatory cells (T and B cells, myeloid, and natural killer cells) to the islets leading to insulitis (1, 2).

The vagus nerve innervates the pancreas by the intrapancreatic parasympathetic nerve endings where the neurotransmitter, acetylcholine (ACh) is released. ACh, in turn, can bind to the nAChRs and mAChRs expressed on pancreatic cells and hence play a key role in regulating pancreatic metabolic functions including glucose homeostasis (3). Increased vagal activity induces insulin secretion by acting on mAChRs expressed on the pancreatic β-cells (4–8). Although β-cells seem to express several subtypes of muscarinic receptors (9–11), M3 mAChR is the most abundant on these cells (12–14) and the one that mediates insulin release (15). Mice selectively lacking M3 mAChR in pancreatic β-cells have an impaired glucose tolerance and a significantly reduced insulin release. In contrast, mice overexpressing pancreatic M3 mAChR exhibit an increase in glucose tolerance and insulin secretion (16). There is also evidence that pancreatic β-cells functionally express different subunits (α4, α5, α7, and β2) of nAChRs (17) but the involvement of these receptors in β-cell function is still controversial. While some studies reported no effect of nAChR agonists on hyperglycemia or β-cell function (17–19), other studies showed that the administration of specific α7nAChR agonists reduced hyperglycemia in diabetic animal models (20–22).

The vagus nerve also serves as a link between the central nervous system and the immune system through the cholinergic anti-inflammatory pathway where ACh suppresses the release of pro-inflammatory cytokines (TNFα, IL-6, HMGB1), attenuating the inflammatory response in sepsis and inflammatory diseases (23–27). Specifically, the α7nAChR has been reported to have a critical role in the inhibition of pro-inflammatory cytokine production by macrophages as well as in other immune mechanisms like apoptosis of T cells and suppressive function of T regulatory cells (28). Furthermore, the presence of a cholinergic system in non-neuronal cells, including immunocompetent cells, has been extensively demonstrated. These cells have choline acetyltransferase (Chat) and acetylcholinesterase (AChE) enzymes as well as choline transporters needed for ACh production (29). Additionally, immune cells express both muscarinic and nicotinic ACh receptors (26, 29–32), indicating that the cholinergic system may be involved in the regulation of the immune response. The α7nAChR is expressed on neutrophils, macrophages, B and T cells, and dendritic cells as well as on enterocytes, endothelial and microglial cells (26, 28, 33, 34), and has been implicated in the pathogenesis of autoimmune diseases (28).

We and others previously demonstrated that activation of the cholinergic nervous system through the administration of specific acetylcholinesterase inhibitors (AChEI) attenuates the development of hyperglycemia and experimental diabetes (36, 37). In our model system, prophylactic cholinergic stimulation induced by paraoxon, an irreversible AChEI, prevented the incidence and development of STZ-mediated hyperglycemia and type-1 diabetes in the multiple low-dose streptozotocin (MLD-STZ) mouse model (36). This correlated with a reduction in T cell infiltration into pancreatic islets, preservation of the structure and functionality of β-cells, and a reduction in pro-inflammatory cytokines. The present study was designed to assess the effect of mAChRs and nAChRs antagonists, mecamylamine hydrochloride and atropine, respectively, on AChEI-mediated prevention of hyperglycemia in the MLD-STZ model. Mecamylamine hydrochloride is a non-selective antagonist of nAChRs (specially α3β4, α4β2, α3β2, and α7) that binds non-competitively to the allosteric site of the receptor and prevents its activation (38). At the neuromuscular junction, mecamylamine is an open-channel blocker (39). At low doses it is used for treatment of various neuro-psychiatric disorders, while at higher doses is indicated for treatment of severe hypertension (40). Atropine is a non-selective, clinically relevant, antagonist of mAChRs that binds competitively to the active site of the receptor without activating it, and completely inhibits the M1, M2, M3, M4, and M5 ACh receptors. Additionally, atropine can block nicotinic α9-containing AChRs (α9^*^nAChRs) (41). Both mecamylamine and atropine cross the blood-brain barrier. Our results indicate that inhibition of nAChR did not abolish the anti-diabetic effect of AChEI. In contrast, inhibition of mAChRs largely reversed the beneficial effect of paraoxon and was associated with increased islet cell infiltration and β-cell damage. These findings suggest a model in which mAChRs can influence the outcome of inflammatory autoimmune diseases.

## Materials and Methods

### Experimental Animals

C57BL/6 male mice were purchased from the Jackson Laboratory (Bar Harbor, ME, USA) and bred in the animal facility of the College of Medicine and Health Sciences, UAE University. Female mice aged 8–10 weeks (weight range 20–22 g) were used for the experiments. All studies involving animals were carried out in accordance with, and after approval of the animal research ethics committee of the College of Medicine and Health Sciences, UAE University.

### Chemicals

Paraoxon (Sigma, St. Louis, MO, USA), an organophosphorus compound, is a highly specific, irreversible, inhibitor of AChE (AChEI). The preparation for paraoxon administration has been described in detail (42). A working solution for intraperitoneal (i.p.) injection was prepared *ex tempore* in pyrogen-free saline to a concentration of 80 nmol/ml. Each mouse received 40 nmol/day of AChEI or saline as control. Atropine sulfate (10 mg/kg) and mecamylamine hydrochloride (MCA; 2 mg/kg), both from Sigma, were injected i.p. 15 min prior to paraxon injection in a volume of 100 μl/day/mouse. These doses were chosen to be in the pharmacological range based on abundant evidence from the literature (43–46). Streptozotozin (STZ; Sigma) was prepared *ex tempore* in citrate buffer (pH 4.5) and used i.p. at 60 mg/kg/day per mouse.

### Diabetes Induction

The protocol for diabetes induction has been described (36). Mice received five daily doses of STZ; control mice received citrate buffer. At different time points post-STZ administration, blood was drawn from the tail vein to determine glucose levels using *One-Touch-ultra-strip* (Lifescan, Zurich, Switzerland). Hyperglycemia was defined as a non-fasting blood glucose level of >200 mg/dl.

### Experimental Protocol

Twenty-five age-matched mice were randomly assigned into five groups (3–5 mice per group). Group I received daily i.p. injection of sterile saline. Group II received daily injection of AChEI. Group III received MCA and AChEI daily injections. Group IV was daily injected with atropine and AChEI. All treatments lasted for 3 weeks (5 day/week). Mice were weighed weekly, at which time blood was collected and analyzed for AChE activity. At the end of treatment, group I was divided into 2 subgroups with 3–5 mice/group, A and B. Group IA (Saline) received daily injections of citrate buffer while groups IB (Saline+STZ), II (AChEI+STZ), III (MCA+AChEI+STZ), and IV (Atropine+AChEI+STZ) received daily injection of STZ for 5 consecutive days. Mice were followed for blood glucose level for up to 60 days post-STZ administration at which time they were sacrificed, and pancreatic tissue collected for analysis.

### AChE Activity of Red Blood Cells (RBC)

The detailed procedure for determining AChE enzyme activity in RBC has been described (42, 47). Briefly, freshly drawn venous blood samples were incubated with DTNB (10 mM) and ethopropazine (6 mM) for 20 min at 37°C prior to addition of acetylthiocholine. The change in the absorbance of DTNB was measured at 436 nm. The AChE activity was calculated using an absorption coefficient of TNB^−^ at 436 nm (ε = 10.6 mM^−1^ cm^1^). The values were normalized to the hemoglobin (Hb) content (determined as cyanmethemoglobin) and expressed as mU/μM/Hb enzyme activities were expressed as percentage of the baseline activity (100%).

### Glucose Tolerance Test (GTT)

Mice were fasted for 16 h, but with free access to water. Blood was obtained from the tail-vein and assessed for baseline fasting glucose levels using a One-touch Ultra glucometer. Mice were then weighed and received 2 g/kg body weight of glucose by i.p. injection (30% glucose solution). Blood samples were subsequently collected at 10, 20, 60, and 120 min to determine glucose levels.

### Histology and Immunohistochemistry of Pancreatic Tissue

The histological analysis of excised pancreatic tissue was performed following a previously described protocol (48, 49). Tissue sections were stained with haematoxylin and eosin (H&E) and images were captured using Olympus BX51 microscope equipped with digital camera DP26 (Tokyo, Japan). Indirect immunostaining for insulin was performed using guinea pig polyclonal antibody (Dako, Carpinteria, CA, USA) followed by FITC-conjugated donkey anti-guinea pig IgG (Jackson ImmunoResearch, West Grove, PA, USA). Slides were counter-stained with propidium iodide (BD Biosciences, USA) and then examined and photographed under a Nikon C1 laser scanning confocal microscope.

### Quantitative RT-PCR

qRT-PCR was carried out as previously described (50) on RNA extracted from pancreatic tissue of each animal. After RNA extraction and purification, cDNA was synthesized using Taqman reverse transcription reagents (Applied Biosystems, Foster City, CA, USA) following manufacturer's protocol. TaqMan primer and probe were used to study the expression of insulin (Applied Biosystems). Transcript levels of target gene were normalized according to the dCq method to respective mRNA levels of the housekeeping gene HPRT.

### Statistical Analysis

Statistical significance between control and treated groups was analyzed using the unpaired, two-tailed Student's *t*-test, using the statistical program of GraphPad Prism version 6 software. For multiple comparisons, we used two-way ANOVA with Tukey's *post-hoc* test (GraphPad Prism). Differences between experimental groups were considered significant when *P*-values were <0.05.

## Results

### Cholinergic Pathway-Induced Protection Is Mediated Primarily Via mAChRs

**Figure 1:**
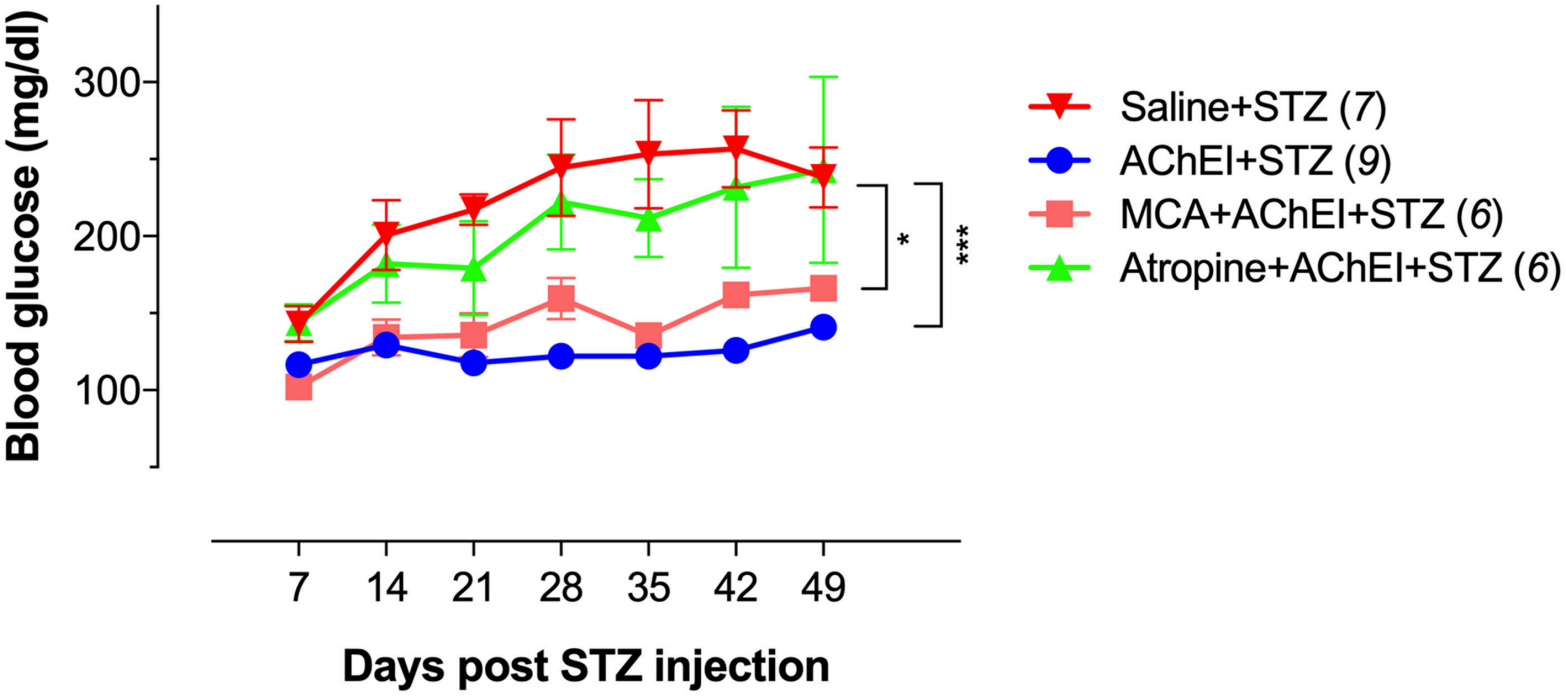
Cholinergic pathway-induced anti-hyperglycemic effect is primarily mediated via mAChRs. Mice were pretreated with saline or paraoxon (AChEI) alone or concurrently with atropine or mecamylamine for 3 weeks followed by STZ for five consecutive days (MLD-STZ). Blood glucose concentrations were measured at the indicated time points. Mice with blood glucose levels >200 mg/dl were considered diabetic. Data are pooled from two independent experiments. The numbers in parenthesis represent the total number of mice per group. For the statistical analysis, the two-way ANOVA with Tukey's multiple comparisons post-test was used. Asterisk denote significance between the saline/STZ group and the indicated experimental groups (*p ≤ 0.05, ***p ≤ 0.001).

As reported earlier (36), MLD-STZ administration in saline-pretreated mice induced progressive hyperglycemia that led to the animals becoming diabetic by the second week of STZ injection (Figure 1; Saline+STZ group). In contrast, paraoxon pretreatment prevented the development of hyperglycemia and diabetes in STZ-treated mice (AChEI+STZ group). When the nAChR antagonist MCA was administered together with paraoxon, transient elevation in blood glucose levels were observed in 2 out of 6 mice on day 28 post STZ treatment but these levels normalized thereafter (MCA+AChEI+STZ group). In contrast, when the mAChR antagonist atropine was co-administered with paraoxon, STZ treatment induced a progressive increase in blood glucose averaging >200 mg/dl by day 28 post-STZ treatment. These elevated levels persisted in 4 out of 6 mice in this group until the end of the observation period (Figure 1; Atropine+AChEI+STZ group). These results suggest that the mAChRs play an important role in the AChEI-induced protection in this model.

**Figure 2:**
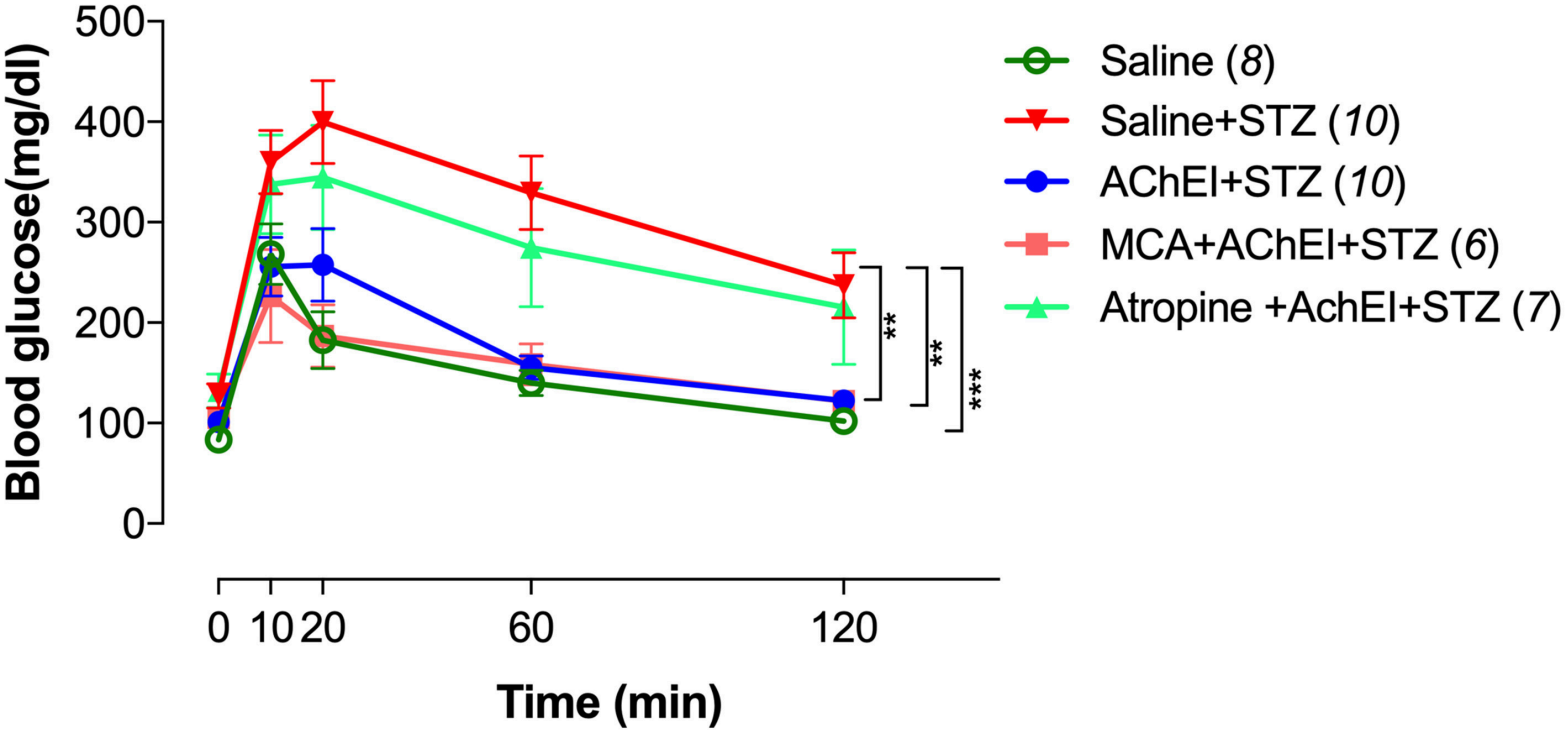
AChEI improves glucose tolerance test (GTT). Animals were pretreated with saline or AChEI alone or concurrently with atropine or mecamylamine for 3 weeks followed by MLD-STZ. At day 56 post-STZ administration, GTT was performed and blood glucose response recorded. Data are shown as comparative responses between saline controls, saline+STZ, AChEI+STZ, MCA+AChEI+STZ, and Atropine+AChEI+STZ treated mice. Values represent mean ± SEM of 6–10 mice per group from two independent experiments. The numbers in parenthesis represent the total number of mice per group. For the statistical analysis, the two-way ANOVA with Tukey's multiple comparisons post-test was used. Asterisk denote significance between the saline/STZ group and the indicated experimental groups (**p ≤ 0.01, ***p ≤ 0.001).

Mice in the different groups were subjected to the GTT to evaluate their response to i.p. injected glucose (2 g/kg body weight) following a fasting period of 16 h (Figure 2). Significantly higher levels of blood glucose were observed in saline+STZ group compared to saline control. In the saline control group, the maximum glucose level was reached after 10 min (268 mg/dl), sharply decreased by 20 min (182 mg/dl), and gradually normalized (102 mg/dl) by the end of the test (120 min). In contrast, STZ-treated mice reached a maximum level of 400 mg/dl after 20 min and, despite slowly decreasing over time, remained significantly elevated (237 mg/dl) at the end of the test period.

Mice receiving paraoxon (AChEI)+STZ exhibited an almost identical response in the IPGTT (intraperitoneal GTT) to normal controls. Importantly, these results show that paraoxon pretreatment not only prevented the development of diabetes but also rendered the animals able to respond normally to high levels of glucose.

In the experimental MCA+AChEI+STZ animal group, blocking of nAChRs failed to alter the protective effect of paraoxon as mice were able to control the increase in blood glucose levels in a manner indistinguishable from control mice. In sharp contrast, animals in the atropine+AChEI+STZ group exhibited response kinetics that were very similar to the saline+STZ group, indicating that blocking of mAChRs mitigated the protection afforded by paraoxon.

### Abrogation of ACh-Mediated Protection by a Muscarinic Receptor Antagonist

**Figure 3:**
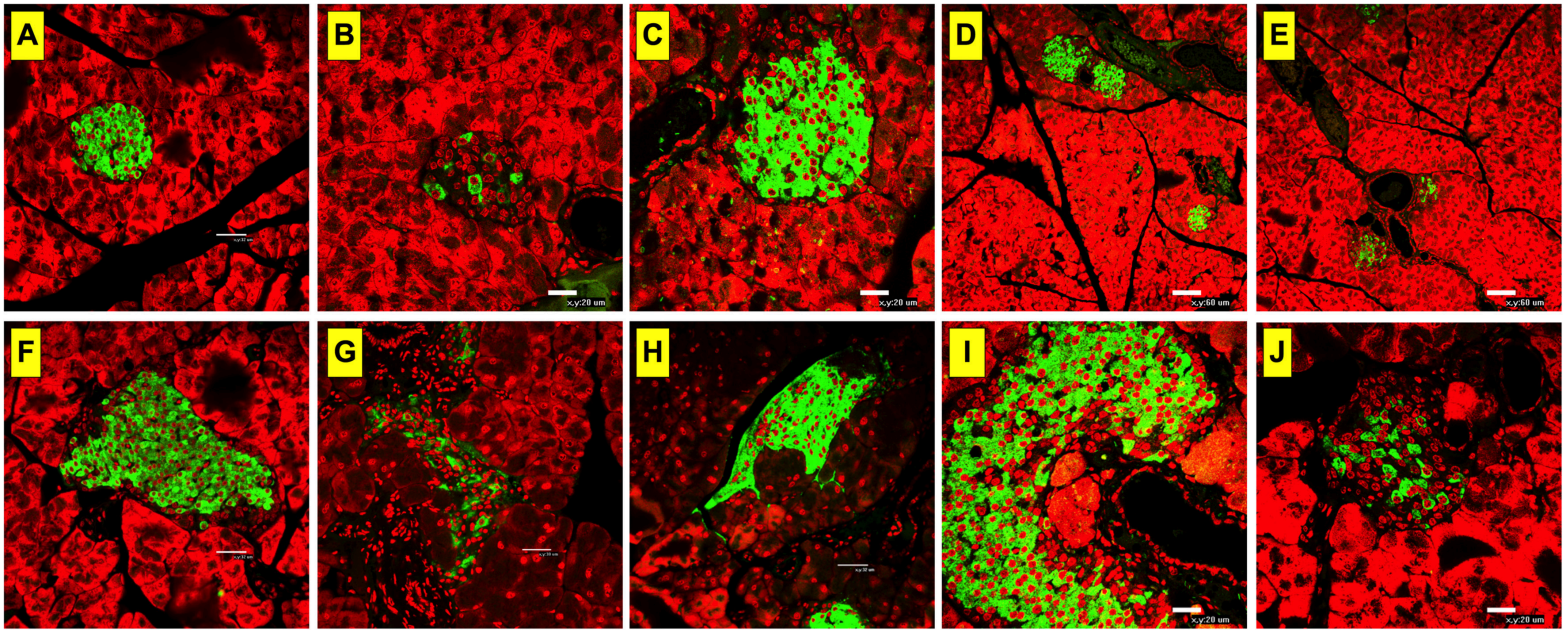
Insulin production is mostly modulated by muscarinic ACh receptors. Mice were treated with AChEI or saline for 3 weeks and then challenged with STZ. At day 60 post STZ administration mice were sacrificed, pancreas excised, fixed, and stained with insulin-specific antibody. Light confocal micrographs of insulin-expressing β-cells in pancreatic islets of mice treated with only saline **(A,F)**, saline+STZ **(B,G)**, AChEI+STZ **(C,H**), MCA+AChEI+STZ **(D,I)**, and Atropine+AChEI+STZ **(E,J)** are shown. Bars in the figures indicate 20 μm **(B,C,I,J)**, 32 μm **(A,F,G,H)**, and 60 μm **(D,E)**. Photos are representative of two individual experiments (*n* = 4 mice/group).

Immunohistochemical analysis of insulin expression was performed on pancreatic tissue at day 60 post-STZ administration. Consistent with our previous findings (36), STZ administration induced loss of insulin in the islets of Langerhans in STZ-injected group (saline + STZ) (Figures 3B,G) compared to normal controls (Figures 3A,F). In contrast, paraoxon pre-treated mice (AChEI + STZ) exhibited intact islets, well preserved from the destructive effect of STZ on insulin producing cells (Figures 3C,H). When paraoxon treatment was administered together with MCA to block nAChRs (MCA+AChEI+STZ group), the protective effect of paraoxon over the pancreatic β cells was mostly preserved (Figures 3D,I). However, administration of paraoxon with atropine (atropine+AChEI+STZ) led to a dramatic decrease in insulin expression, indicating that blocking of the mAChRs ameliorated the protective effect of AChEI on insulin production in the islets (Figures 3E,J).

**Figure 4:**
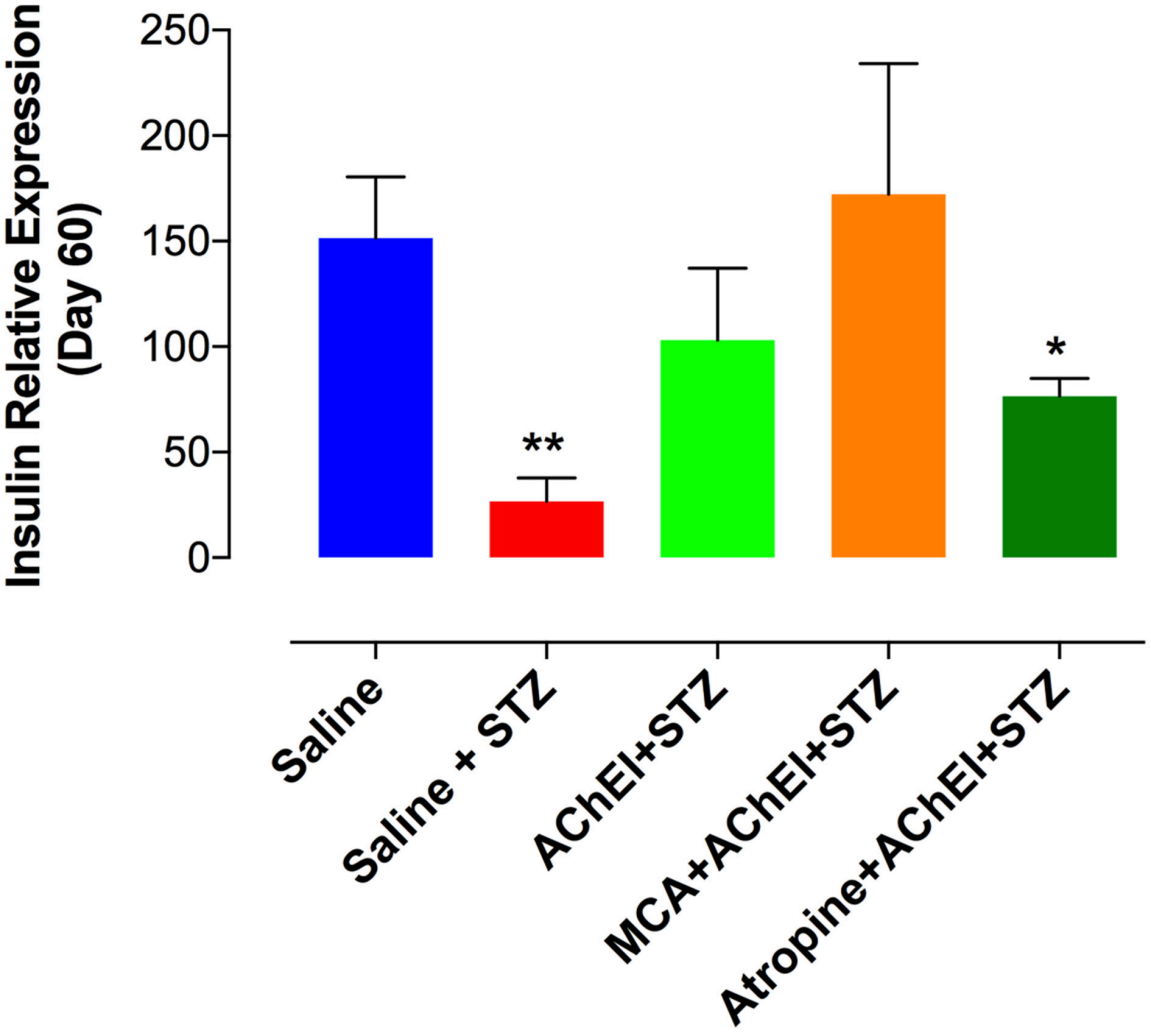
Insulin expression in pancreatic tissue. Mice were treated as described in Figure 2 legend. At day 60 post STZ administration, pancreatic tissue was obtained and processed for RNA extraction. mRNA expression of pancreatic insulin was determined by qRT-PCR. The graph depicts values from two independent experiments (*n* = 8–10 mice/group). Asterisks indicate significance compared to the saline control group (*p ≤ 0.05, **p ≤ 0.01).

We also analyzed the insulin mRNA levels in pancreatic tissue at day 60 post-STZ administration (Figure 4). STZ treatment in saline group (saline+STZ) led to a 6.3-fold reduction in the level of insulin mRNA relative to control saline. Similar levels of insulin mRNA to the saline control group were found in paraoxon pretreated (AChEI+STZ) and MCA+AChEI+STZ groups. When atropine was injected with the AChEI, a significant reduction in the levels of insulin mRNA was observed, compared to saline control group. It is worthy to note that although no significant differences were obvious among the three paraoxon treated groups, the levels of insulin mRNA were higher in the MCA+AChEI+STZ than in AChEI+STZ and Atropine+AChEI+STZ groups which could indicate the importance of the muscarinic ACh receptors in the production of insulin.

### Blockade of mAChRs Increases Cellular Infiltration and Leads to Islet Destruction

**Figure 5:**
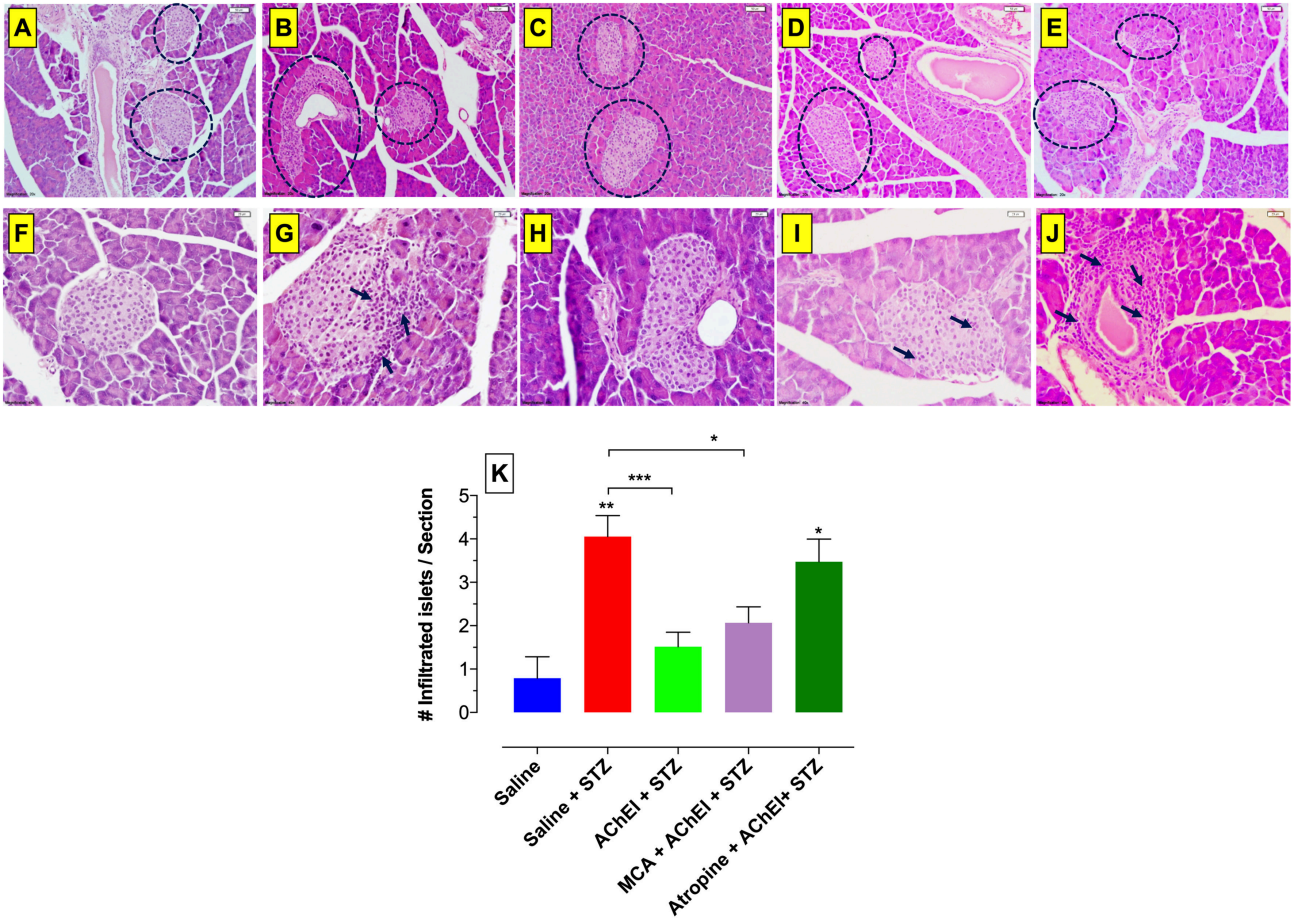
Loss of islets morphology following blockade of mAChRs. H&E staining of paraffin embedded pancreata from mice sacrificed at day 60 post-STZ administration. Five experimental groups are represented in this figure: saline **(A,F)**, saline+STZ **(B,G)**, AChEI+STZ **(C,H**), MCA+AChEI+STZ **(D,I)**, and Atropin+AChEI+STZ **(E,J)**. Dashed lines delineate Langerhan's islets and arrows indicate inflammatory infiltrates. Images are representative of two independent experiments (*n* = 4 mice/group). The bar in the figures indicates 50 μm (panels **A–E**) and 20 μm (panels **F–J**). **(K)** Quantitative estimation of infiltrated islets. H&E-stained pancreatic sections were examined to quantify the number of islets containing inflammatory cell infiltrates. The graph depicts values from two independent experiments (*n* = 4 mice/group/experiment). Three sections per mouse were used for the quantification. Asterisks denote significance between the indicated experimental groups (*p ≤ 0.05, **p ≤ 0.01, ***p ≤ 0.001). Asterisks on top of the bars indicate significance with the saline group.

Pancreatic tissue stained with hematoxylin and eosin was analyzed at day 60 post-STZ administration. Saline control mice had intact islets with no visible inflammatory infiltration (Figures 5A,F). STZ administration to saline-pretreated mice induced a massive infiltration of inflammatory cells that disturbed the normal morphology of the islets (Figures 5B,G). In contrast, paraoxon treatment prior to STZ administration largely prevented any significant inflammatory cell infiltration and showed intact islet morphology (Figures 5C,H). Likewise, cotreatment of paraoxon and mecamylamine previous to STZ administration prevented islet infiltration by inflammatory cells and preserved their normal structure (Figures 5D,I). In contrast, pretreatment with paraoxon and atropine led to extensive cellular infiltration into the islets and destruction of normal morphology (Figures 5E,J). It should be noted that in the latter group, a few healthy islets could still be observed in some mice. These results suggest that the presence of active mAChRs is essential to protect β-cells against STZ-mediated destruction. It is worth noting that mice treated with AChEI alone show no evidence of any alterations in islet morphology or extent of cellular infiltration (36) and, hence, this experimental group was not included in the current study.

Next, we sought to quantitate the observed alterations in islet morphology by determining the number of islets with infiltrated cells (Figure 5K) in H&E stained-pancreatic sections of each experimental group. Compared to saline controls, STZ treatment resulted in highly infiltrated islets. Pretreatment with AChEI led to a significant reduction (>65%) in the extent of cellular infiltrates into the pancreatic islets, hence preserving islet morphology. The number of islets with lymphoid infiltration in the AChEI+MCA+STZ experimental group was comparable to the AChEI+STZ group. However, blocking of the mAChRs with atropine led to a significant increase in the number of infiltrated islets to a level comparable to that observed in the saline+STZ group, indicating the need for functional mAChRs to avoid inflammation and, therefore, maintain healthy insulin-producing β cells.

## Discussion

It is well-established that ACh regulates the endocrine function of the pancreas and is essential to maintain glucose homeostasis. In rodent's pancreas, the only source of ACh are the neural terminals, in contrast to pancreas of humans where glucagon-producing α-cells are able to produce ACh to modulate β-cell function (3). It has been postulated that the development of autoimmune diabetes (T1D) and other autoimmune diseases is triggered by a dysfunction of the autonomic nervous system (51–53) followed by recruitment of inflammatory cells (22). Failure or insufficient efferent vagus nerve cholinergic output might allow the overproduction of inflammatory cytokines and, therefore, a dysfunctional immune and metabolic regulation that in normal conditions would not occur (51, 52). Our previous work demonstrated that increased cholinergic pathway activation through a chronic administration of paraoxon, an irreversible specific inhibitor of AChE, protected against development of T1D in MLD-STZ mouse model (36). ACh signals through two different types of receptor, muscarinic and nicotinic, both present on pancreatic and immune cells. Our aim in this study was to delineate the role of muscarinic (mAChRs) and nicotinic (nAChRs) receptors in paraoxon-mediated protection using two different receptor antagonists, namely mecamylamine and atropine. Mecamylamine is a non-selective antagonist of nAChRs that binds non-competitively to the allosteric site of the receptor and prevents its activation. Atropine is an antagonist of mAChRs that binds competitively to the active site of the receptor without activating it. Both mecamylamine and atropine can also cross the blood-brain barrier.

In this study, we demonstrate that co-treatment with AChEI and mecamylamine did not abolish the protective effect of AChEI on the development of STZ-mediated hyperglycemia. In contrast, blocking of mAChRs with atropine reversed the protective effect of paraoxon. These results correlated with the immunohistochemical and insulin mRNA analysis of pancreatic tissue where the concurrent administration of mecamylamine, but not atropine, preserved insulin production in β-cells. The fact that insulin production was decreased when mAChRs were specifically inhibited suggests an important role for these receptors in regulating insulin production. The results of the GTT offer further confirmation for this conclusion. Animals in the MCA+AChEI+STZ group, where mAChRs were available, performed better than their counterparts treated with atropine+AChEI+STZ, where mAChRs were blocked, demonstrating the importance of the pancreatic islets' cholinergic innervation in the prevention of diabetes.

It is worth noting the existence of α9^*^ nAChR, a special member of AChRs with a mixed nicotinic and muscarinic profile (41). Originally discovered in cochlear cells (54), α9^*^ nAChRs are now known to be expressed on several cell types including lymphoid and myeloid cells (55–57). The α9^*^ nAChRs are blocked by nicotine (an agonist for the rest of the subunits of nicotinic receptors) and by atropine, a non-selective muscarinic receptor antagonist (41). The involvement of α9^*^ nAChRs in inflammation has been highlighted by the findings that mice genetically deficient in α9 subunit are protected against the development of murine experimental autoimmune encephalomyelitis, a model of multiple sclerosis (58). In the current study, concurrent treatment with atropine plus AChEI abrogated the latter's protective effect against STZ-induced hyperglycemia. This suggests the importance of muscarinic receptors, α9^*^ nAChRs or both in AChEI-mediated protection. In contrast, administration of the general nAChRs antagonist MCA (41, 59) had no effect on AChEI-mediated protection, suggesting that nAChRs may not be important for the beneficial effect. Overall, our findings suggest that muscarinic cholinergic receptors play a predominant role in AChEI-mediated protection in the MLD-STZ model.

STZ is known to induce apoptosis in insulin-producing β-cells (60). Production of AChE by apoptotic cells (61, 62) and the protective effect that AChE inhibitors exert against apoptosis has been well-documented (63). Moreover, there is evidence that ACh acts through mAChRs to protect neuronal cells against apoptosis induced by different stimuli, including pro-inflammaory cytokines and pro-apoptotic agents (64–66). In our model system, paraoxon appears to counteract the pro-apoptotic effect of STZ on β cells, hence inhibiting the development of hyperglycemia and autoimmune diabetes (36). The current findings implicate the mAChRs in this protection, thus demonstrating for the first time the important role played by these receptors in preserving β-cell function.

In summary, we have demonstrated that co-administration of paraoxon and mAChR antagonist blocks the protective signaling of ACh, suggesting a critical role for mAChRs in the protection of β-cells against STZ-mediated death. It is also noteworthy that signaling through mAChRs may act directly to stimulate the secretion of insulin by β cells. Therefore, available evidence suggests that signaling through mAChRs on β cells could act via two different pathways to increase insulin secretion and protect against apoptosis. Additionally, it is important to note that the administration of muscarinic or nicotinic antagonists may affect the receptors present in many different peripheral tissues as well as the brain. The observed *in vivo* effects are likely the result of the sum of multiple activities that involve different tissues, including lymphoid cells. For example, the cholinergic anti-inflammatory pathway, operating at the level of the spleen through the nAChRs, may also limit the inflammatory response in the islets. Although inflammation is considered as a major pathogenic factor in diabetes and its complications, the origin of this inflammation is still unclear (53). It is known that the autonomic nervous system has a role in inflammation and autoimmunity. The cholinergic anti-inflammatory pathway can modulate the inflammatory response to infection and injury and downregulate the production of inflammatory cytokines (24, 67). In a study using NOD mice, it was demonstrated that the neurons innervating pancreatic β-cells were lost before any damage in the islets could be detected (68). Therefore, our results are consistent with the idea that a dysfunction of the autonomic nervous system could be the initiating trigger in autoimmune diabetes.

**Figure 6:**
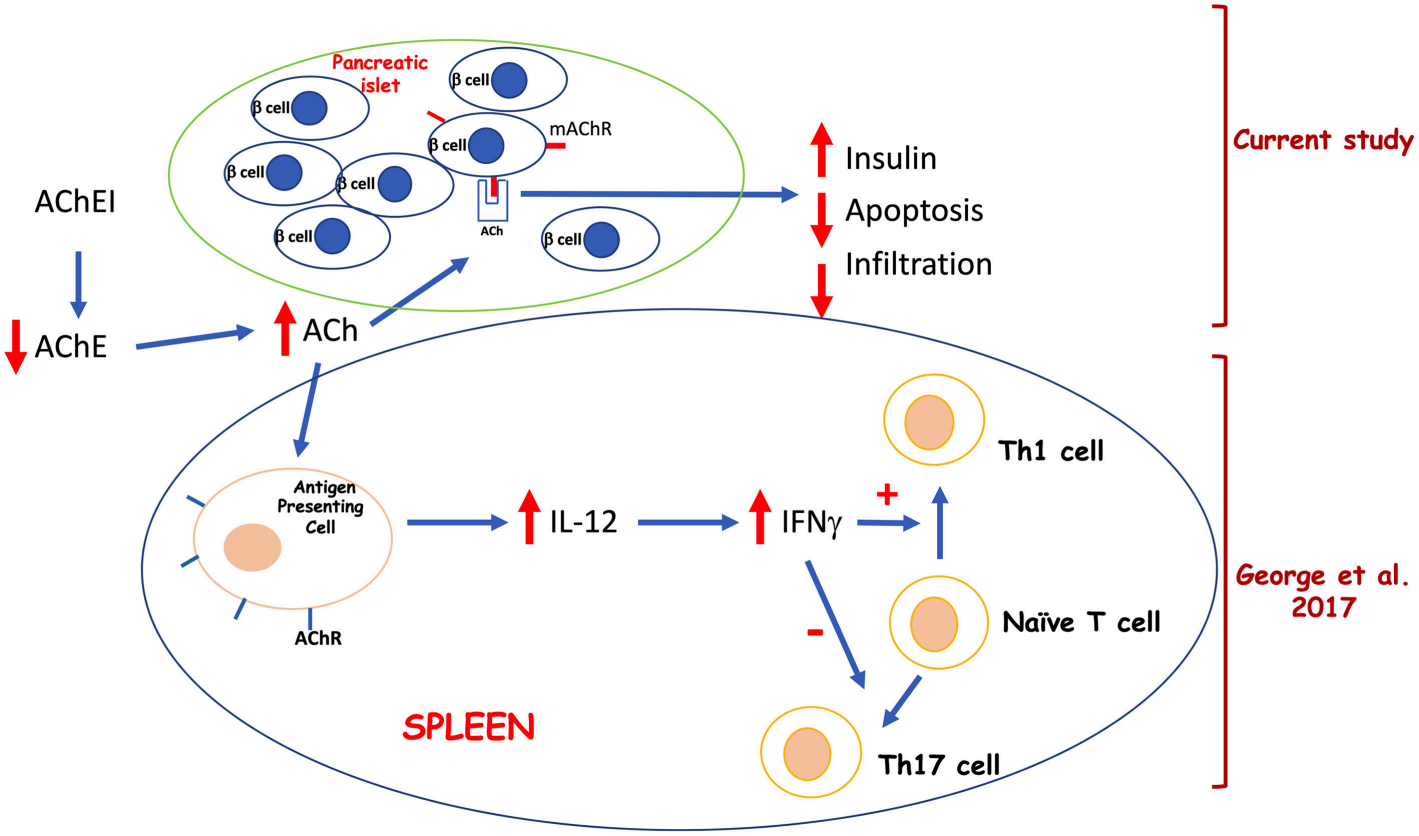
Proposed model for the effects of systemic acetylcholinesterase inhibition on pancreatic β cells and splenic lymphoid cells.

We conclude that the effect of paraoxon treatment on the development of hyperglycemia in the MLD-STZ model is due to a combination of factors: (a) stimulation of insulin production by β-cells and (b) protection against STZ-induced apoptosis, both through the mAChRs, and (c) differentiation of Th1 (T helper 1) cells and mitigation against the development of pathogenic Th17 (T helper 17) cells (36). A diagram summarizing our findings is shown in Figure 6. Our current experimental system could be further probed using a combination of genetic (using M3 AChR-deficient mice) and pharmacologic (using selective M3 AChR antagonists, such as darifenacin) approaches to refine our understanding of the role of the cholinergic pathway in autoimmune diabetes.

## Ethics Statement

All studies involving animals were carried out in accordance with, and after approval of the animal research ethics committee of the College of Medicine and Health Sciences, UAE University.

## Author Contributions

MF-C designed the study, supervised the project, analyzed data, and wrote the final manuscript. JG performed experiments and analyzed data. GB performed histological experiments and analyzed data. YM and AA-M performed all molecular studies. MQ performed biochemical assays for AChE activity and sample processing for histological study. DL and GP contributed to the design of the study. Ba-R designed the study, analyzed data, and revised the final manuscript.

### Conflict of Interest Statement

The authors declare that the research was conducted in the absence of any commercial or financial relationships that could be construed as a potential conflict of interest.

## Abbreviations

ACh: acetylcholine
AChE: acetylcholinesterase
AChEI: acetylcholinesterase inhibitor
AChR: acetylcholine receptor
nAChR: nicotinic acetylcholine receptor
mAChR: muscarinic acetylcholine receptor
MCA: mecamylamine. An “^*^” indicates a set of nAChR that contains the specified subunit but it can contain additional unspecified subunits (35).
